# Regulation of m7G methylation in long COVID: Expression profiles and early predictive value of key genes

**DOI:** 10.1097/MD.0000000000044209

**Published:** 2025-08-29

**Authors:** Wenmei Bai, Fengsen Li

**Affiliations:** a The Fourth Clinical College of Xinjiang Medical University, Urumqi, China; b Department of Respiratory and Critical Care Medicine, Fourth Affiliated Hospital of Xinjiang Medical University, Urumqi, China.

**Keywords:** immunocorrelation analysis, long COVID, N7-methylguanosine, predictive model

## Abstract

Long COVID (LC) poses ongoing public health challenges due to its persistent symptoms following severe acute respiratory syndrome coronavirus 2 infection. Early identification of at-risk individuals remains difficult, and molecular biomarkers are urgently needed. This study aimed to explore the role of N7-methylguanosine (m7G) methylation-related regulatory genes in LC pathogenesis and to develop a predictive model for early detection. Gene expression profiles of LC patients were obtained from the GEO database (GSE224615), and differentially expressed genes (DEGs) were identified. These DEGs were intersected with m7G regulatory genes to identify LC-specific candidates. A protein–protein interaction network was constructed to identify hub genes, and enrichment analyses including Gene Ontology, Kyoto Encyclopedia of Genes and Genomes, and gene set enrichment analysis were performed to investigate the biological relevance of the identified genes. Immune cell infiltration analyses were conducted to explore the immunological features associated with candidate genes. Findings were validated using an external dataset (GSE217948). A clinical prediction model was constructed using Least absolute shrinkage and selection operator regression followed by logistic regression, and evaluated via receiver operating characteristic curve, calibration, and decision curve analysis. A total of 65 DEGs were identified in LC patients, comprising 44 up-regulated and 21 down-regulated genes. Thirty genes overlapped with the m7G regulatory gene set. Functional enrichment revealed significant involvement in pathways such as FceRI-mediated NF-κB activation and platelet aggregation. Correlation analysis showed that several m7G-related genes were associated with altered immune cell infiltration patterns. The external dataset confirmed the reproducibility of gene expression trends. Seven core genes were ultimately selected to build the predictive model, which demonstrated robust performance in distinguishing LC patients from controls. This study highlights the importance of m7G methylation in LC pathogenesis and uncovers novel immune-related mechanisms underlying its persistence. The predictive model based on m7G-related markers provides a promising tool for early LC identification and may inform future diagnostic and therapeutic strategies.

## 1. Introduction

Long COVID (LC), widely acknowledged within the medical community, “LC” describes a constellation of persistent symptoms that individuals endure following infection with the severe acute respiratory syndrome coronavirus 2 (SARS-CoV-2).^[1]^ These enduring symptoms affect multiple organ systems, persist over an extended period, and profoundly impact the quality of life for survivors of COVID-19. The spectrum of symptoms encompasses severe fatigue, respiratory distress, cognitive impairment, anxiety, emotional disturbances, and may extend to encompass headaches, and sleep disorders.^[2–4]^ LC represents a formidable health challenge that transcends age and the severity of the initial infection. Moreover, it continues to pose a substantial and enduring public health threat, persisting for nearly 4 years since its emergence. This issue imposes a substantial burden on both society and healthcare systems, as a significant number of survivors grapple with persistent symptoms that severely curtail their functional capabilities and daily activities.^[5,6]^ Unfortunately, our understanding of LC is still very limited, and the specific pathogenesis is still unclear.

Methylation alterations, in conjunction with associated enzymes, wield a central influence on the infection of host cells by viruses.^[7]^ Notably, distinctive patterns of N6-methyladenosine (m6A) modifications have been discerned in the transcription of a spectrum of DNA viruses, such as Kaposi sarcoma-associated herpesvirus, hepatitis B virus, Epstein–Barr virus, as well as RNA viruses like SARS-CoV-2, human immunodeficiency virus type 1, and rotaviruses.^[8,9]^ N7-methylguanosine (m7G) constitutes a ubiquitous RNA modification, with its initial documentation dating back to the 1970s. This modification is prominently situated at the 5’ caps of eukaryotic mRNA, but is also discernible within the interior of ribosomal RNA and transfer RNA in organisms spanning the biological spectrum.^[10]^ The m7G modification exerts a profound impact on RNA processing, metabolism, and functional attributes. Historically, m7G methylation was primarily attributed to the N7 position of the 5’ terminal guanosine within eukaryotic mRNA, thereby influencing processes such as transcription elongation, mRNA splicing, nucleation, and translation.^[11,12]^ However, contemporary research has unveiled that m7G modifications are not confined solely to mRNA, they are also encountered at internal locations within diverse RNA categories, including transfer RNA, ribosomal RNA, and mRNA.^[12–14]^ Hence, it is a plausible hypothesis that individuals suffering from LC may manifest anomalies in the m7G methylation process.^[15,16]^

What characterizes the m7G methylation modification in LC patients, and which pivotal regulatory genes are involved? This study seeks to elucidate the expression patterns of key regulatory factors in m7G methylation in LC patients and employ these critical regulatory genes to formulate a clinical predictive model for LC. From this innovative methylation perspective, we aim to probe potential mechanisms underlying the onset of LC and provide potential predictive tools for the early clinical identification of this condition.

## 2. Methodology

### 2.1. Data sources

In the course of our investigation, we accessed data pertaining to “LC” from the Gene Expression Omnibus database (GEO) available at http://www.ncbi.nlm.nih.gov/geo/. Subsequently, we identified a pertinent microarray dataset denoted as GSE224615. GSE224615 consisted of a total of 36 samples, encompassing 13 non-long COVID (non-LC) samples and 23 LC samples. To further substantiate our findings, we incorporated an additional dataset, GSE217948, which encompassed a more extensive collection of samples.^[17,18]^ This dataset comprised 334 individuals who tested positive for COVID-19 infection, along with 62 control samples. In addition to the gene expression data, our research encompassed the integration of regulatory genes associated with m7G methylation. These genes were obtained from the m6A2Target database.^[19]^ All data retrieval, cohort selection, bioinformatic processing, and integrative analyses were performed during the period May 2024 to December 2024, following FAIR (Findable, Accessible, Interoperable, Reusable) data principles.

### 2.2. Quality control and screening for differential genes in patients with LC

We conducted data processing using the R software (version 3.6.2; R Foundation for Statistical Computing, Vienna, Austria). To evaluate the integrity of the curated dataset, we employed various techniques, including the examination of box plots, Partial Least Squares Discriminant Analysis (PLS-DA) plots, and hierarchical clustering dendrograms. Our criteria for filtering differential genes were as follows: *P*-value<.05 and |logFC (fold change)|>1. Subsequently, we harnessed the capabilities of the ggplot2 package (version 3.3.3) and ComplexHeatmap package (version 2.2.0) in R to generate volcano plots and heatmaps, providing a comprehensive visual representation of the differential gene expression patterns in LC patients.

### 2.3. Key regulatory factors of m7G methylation in patients with LC

We conducted an intersection analysis between the differentially expressed genes observed in LC patients and genes associated with m7G methylation regulation. Following this, we constructed a Venn diagram to visually represent the overlap between these gene sets. Additionally, we performed a correlation analysis between the identified genes and the dependent variables using R software, specifically employing the ggplot2 package (version 3.3.6). The outcomes of the analysis were subsequently presented through co-expression heatmaps, lollipop charts, and network diagrams to enhance clarity and facilitate comprehension.

### 2.4. PPI network construction and enrichment analyses

For the purpose of our research, we utilized the STRING database (version 11.0) to establish an intricate protein–protein interaction (PPI) network.^[20]^ To ensure the reliability of our PPI network, we set a stringent threshold, only considering interactions with a combined score >0.7. In this research, we conducted comprehensive enrichment analyses, specifically focusing on Gene Ontology (GO) and Kyoto Encyclopedia of Genes and Genomes (KEGG) pathway enrichment analysis.^[21,22]^ We employed R software in conjunction with the “GO plot” package to execute the GO analysis, which encompassed the assessment of cellular component, biological process, and molecular function. The identified differentially expressed genes (DEGs) were subject to annotation using the clusterProfiler package (version 3.14.3) and subsequent visualization using ggplot2 (version 3.3.3) for both GO and KEGG enrichment analyses. The criteria for establishing statistical significance entailed a *P*-value <.05 and a gene count of 2 or more.

To assess the association between gene sets and biological signals, we conducted Gene Set Enrichment Analysis (GSEA). This GSEA analysis was performed using GSEA_4.1.0 and leveraged c5: GO gene sets (c5.all.v7.1.symbols.gmt). The analysis was conducted within the framework of the clusterProfiler package (version 3.14.3) in R software. Criteria for determining statistical significance in GSEA included a |Normalized Enrichment Score| >1, false discovery rates below 0.05, and a *P*-value <.05.

### 2.5. Correlation analysis of key genes and phenotypes of immunoinfiltrating organisms

This study employs 3 distinct methods, namely Cibersort, GSEA, and Estimate, to compute immune cell scores within key genes and to infer the composition of the immune cell populations.^[18,23,24]^ The procedures are detailed as follows: first, we employ the Cibersort core algorithm, implemented in the CIBERSORT R script. This method utilizes markers for 22 different types of immune cells, which can be accessed on the CIBERSORTx website (https://cibersortx.stanford.edu). It quantifies the extent of immune cell infiltration associated with differential gene expression. Next, we assess the immune infiltration status of the aforementioned data using the ssGSEA algorithm from the R package GSVA. Furthermore, we calculate immune scores by utilizing the algorithm provided by the R package Estimate, which incorporates internal markers. This approach allows for a more comprehensive evaluation of the immune landscape within the context of this study.

### 2.6. Validation and receiver operating characteristic (ROC) curve of key genes

To ensure robust model performance, we assessed its sensitivity and specificity through ROC analysis. Sensitivity, reflecting the model’s ability to correctly identify LC patients, and specificity, representing the model’s ability to distinguish controls from LC cases, were evaluated. This study embarked on the validation of the chosen pivotal genes. We obtained the dataset GSE217948 from the GEO database, which encompasses 334 COVID patients and 62 control subjects. The key genes within the GSE217948 dataset were systematically subjected to validation. We plotted ROC curves for key genes using the ggplot2 software package (version 3.3.3). Statistical significance for differences was established at a significance level of *P*-value<.05.

### 2.7. Prediction model construction

We utilize the independently validated differentially expressed genes as our independent variables, while the presence of LC in an individual serves as the dependent variable. We apply a least absolute shrinkage and selection operator model to identify potential predictive factors. The model’s sensitivity and specificity were assessed using a 10-fold cross-validation approach to ensure the reliability and robustness of the results. Furthermore, decision curve analysis was employed to evaluate the clinical utility of the predictive model. This analysis provides insights into whether the model’s predictions would be beneficial in a real clinical context, considering both the sensitivity and specificity trade-offs at various threshold probabilities.

Following this, we incorporate the identified candidate factors into a logistic regression model to examine their association with LC, ultimately crafting a clinical prediction model. In the final step, we conduct a comprehensive evaluation of the model, utilizing ROC curves, calibration plots, and decision curves.

### 2.8. Statistical analysis

This study utilized statistical analysis with R version 3.6.2 and GraphPad Prism 9 (GraphPad Software, San Diego). Group comparisons were executed via unpaired Student *t* tests, and the diagnostic performance was evaluated using ROC curve analysis. The levels of statistical significance were defined as follows: non-significant (Ns): *P*-value ≥ .05, *significant: *P*-value<.05, **highly significant: *P*-value<.01, ***very highly significant: *P*-value<.001, ****extremely significant: *P*-value<.000.

## 3. Results

### 3.1. Quality control and screening for differential genes in patients with LC

**Figure 1.**
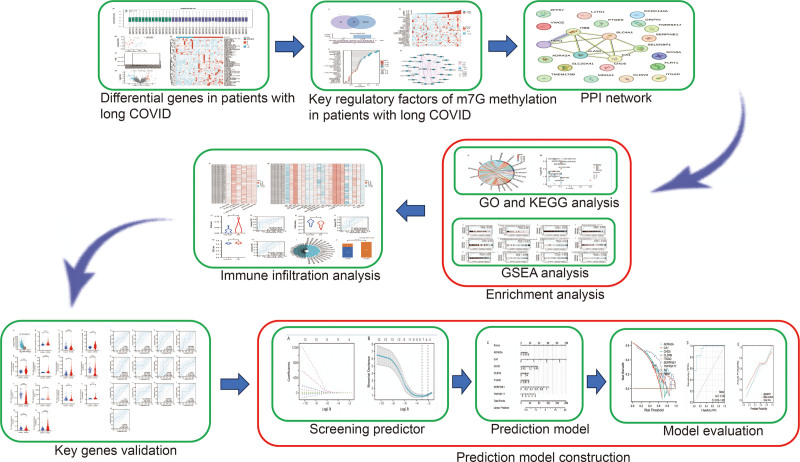
Study workflow. The research methodology for this study involves the following sequential steps: firstly, the identification of m7G methylation regulatory factors associated with long COVID; secondly, the creation of a PPI network and the execution of functional enrichment analysis for pivotal genes; next, an examination of the correlation between these pivotal genes and specific immune cell types; then, the verification of these pivotal genes and the subsequent analysis of ROC curves; ultimately, the development of a predictive model will be undertaken. GO = Gene Ontology, GSEA = gene set enrichment analysis, KEGG = Kyoto Encyclopedia of Genes and Genomes, m7G = N7-methylguanosine, PPI = protein–protein interaction, ROC = receiver operating characteristic.

**Figure 2.**
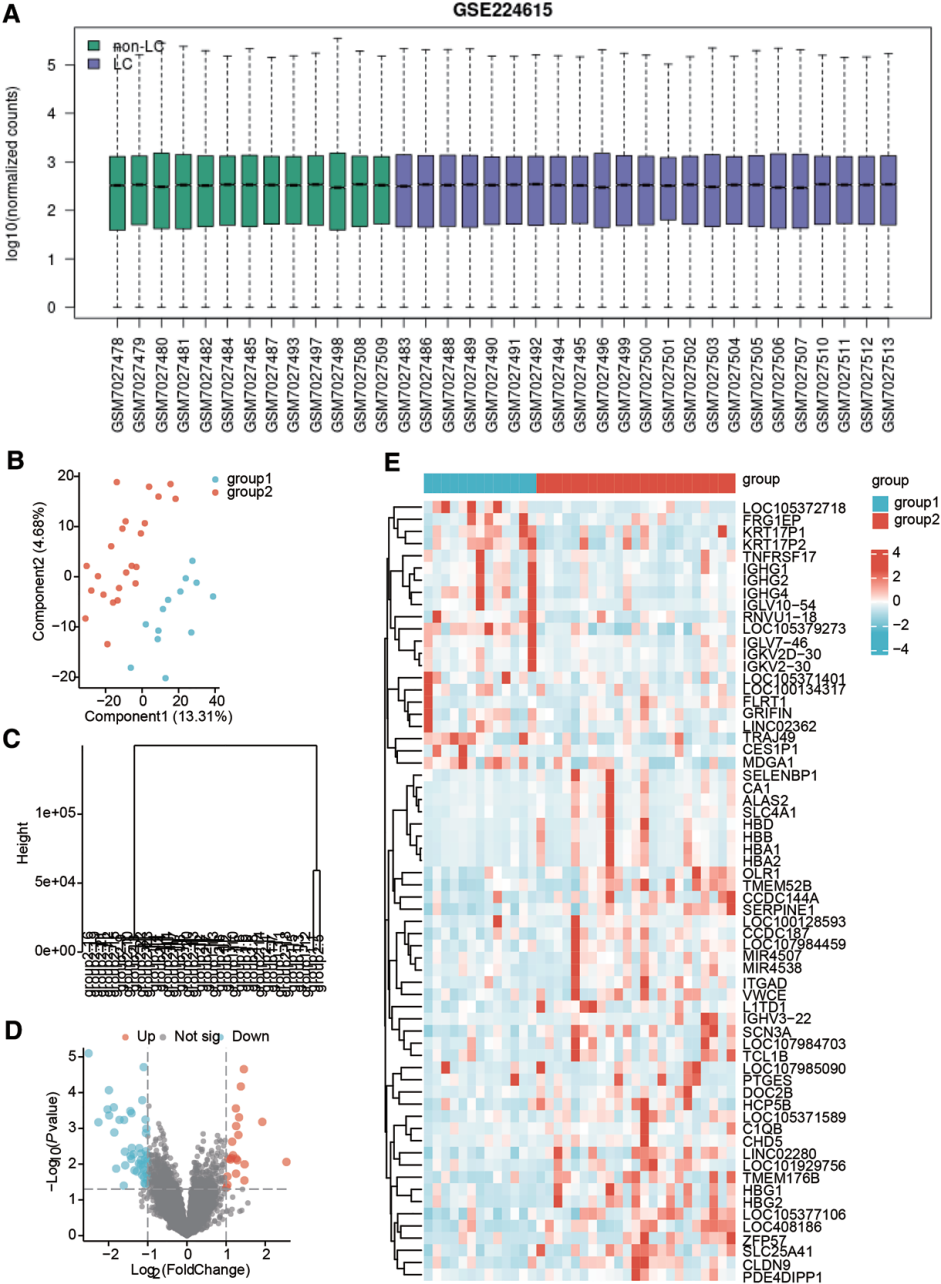
Quality control and screening for differential genes in patients with LC. (A) Boxplot representing data from all samples. (B) PLS-DA plots. (C) Hierarchical clustering dendrograms; the volcano plot (D) and heatmap (E) are utilized to demonstrate the differential gene expression. LC = long COVID, PLS-DA = Partial Least Squares Discriminant Analysis.

Figure 1 illustrates the comprehensive flowchart of our current investigation. The boxplot of processed data demonstrates a close alignment of medians for each sample (Fig. 2A). Both Partial Least Squares Discriminant Analysis (PLS-DA) plots and hierarchical clustering dendrograms provide clear evidence of a distinct separation between LC and control samples (Fig. 2B and C). After procuring data from GSE224615, we identified 65 DEGs, encompassing 44 up-regulated DEGs and 21 down-regulated DEGs. Figure 2D and E portrays the volcano plot and heatmap representing these DEGs.

### 3.2. Key regulatory factors of m7G methylation in patients with LC

**Figure 3.**
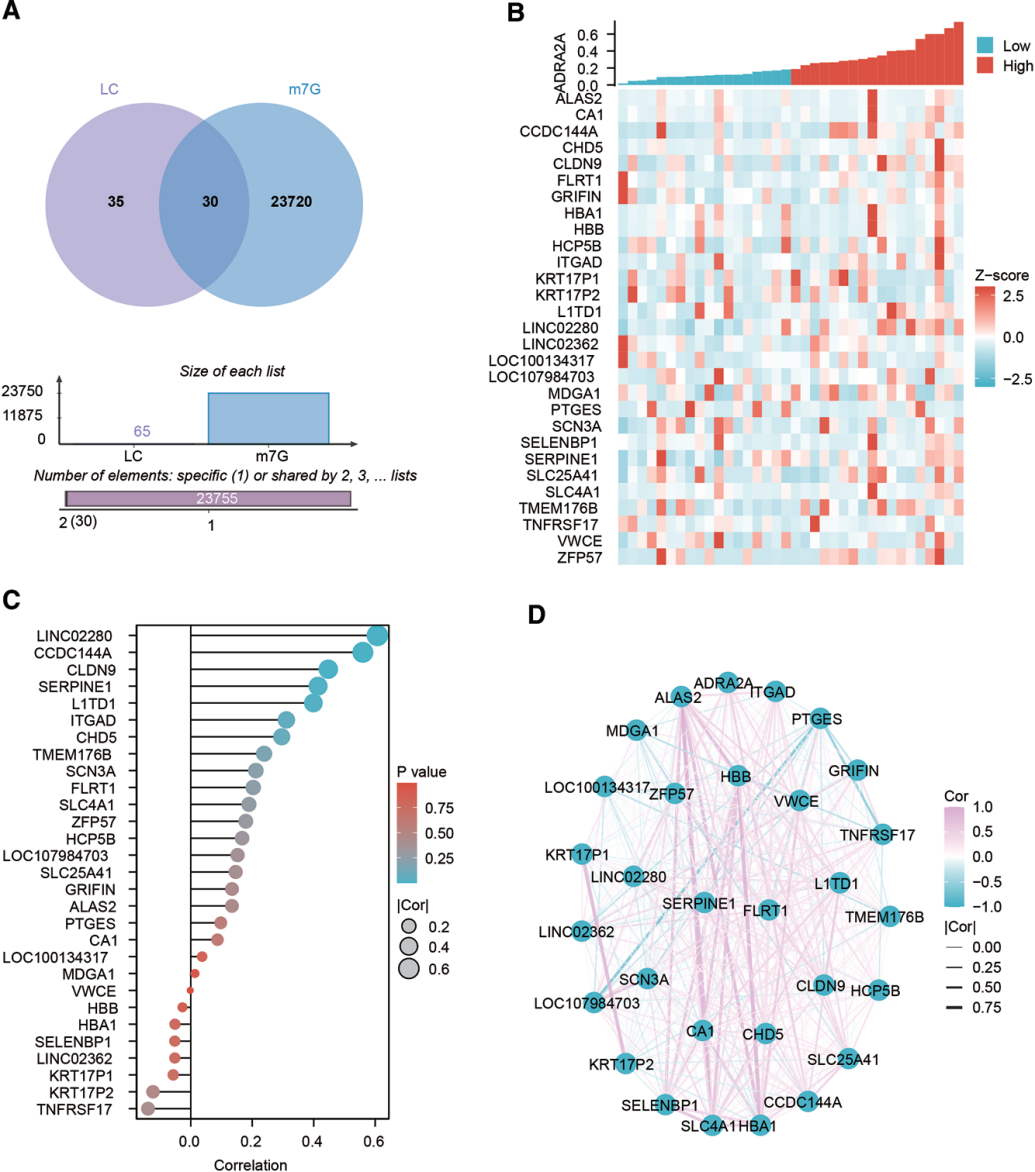
Key regulatory factors of m7G methylation in patients with LC. (A) Illustrated the overlapping outcomes between LC and regulatory factors of m7G methylation using a Venn diagram; employed co-expression heatmaps (B), lollipop charts (C), and network diagrams (D) to meticulously examine the interrelationships among the DEGs. DEGs = differentially expressed genes, LC = long COVID, m7G = N7-methylguanosine.

Moreover, we proceeded to intersect the DEGs associated with LC, with the genes linked to m7G methylation sourced from the m6A2Target database. This analysis resulted in the identification of 30 genes that were common to both datasets, as illustrated in Figure 3A. Within this subset of 30 genes, 23 exhibited an increase in expression, while 7 demonstrated a decrease in expression (Table 1). Following this, we employed co-expression heatmaps, lollipop charts, and network diagrams to scrutinize the interconnections among these DEGs, as depicted in Figure 3B, C, and D, respectively.

**Table 1:** Key regulatory factors of m7G methylation in patients with LC.

Symbol	*P*-value	LfcSE	Stat	Log2FC	Base mean	Type
ADRA2A	.00056855	0.3068	3.44619	1.057319	32.45	Up
ALAS2	.00000798	0.5622	4.465676	2.510518	138.17	Up
TNFRSF17	.03781655	0.4967	-2.07684	-1.03157	32.82	Down
CA1	.00043575	0.5646	3.517428	1.985766	18.96	Up
HBA1	.00025878	0.5113	3.653408	1.86794	9024.31	Up
HBB	.00008561	0.5071	3.928134	1.99182	8498.13	Up
ITGAD	.01461402	0.4469	2.441805	1.091363	49.24	Up
SERPINE1	.00001936	0.2583	4.272123	1.103438	30.08	Up
SCN3A	.00016309	0.3023	3.770246	1.139928	171.24	Up
SLC4A1	.00029477	0.5597	3.619854	2.025888	65.03	Up
SELENBP1	.00718522	0.5285	2.688136	1.420724	29.38	Up
CLDN9	.03379891	0.5119	2.122463	1.086593	6	Up
PTGES	.01079426	0.6181	2.54929	1.575714	147.19	Up
CCDC144A	.01491518	0.5498	2.434432	1.338377	200.87	Up
FLRT1	.00561529	0.3656	2.769439	1.012544	12.03	Up
CHD5	.00723297	0.3982	2.685923	1.069643	32.25	Up
TMEM176B	.01205476	0.4468	2.510537	1.121672	1922.64	Up
L1TD1	.00037666	0.3977	3.555918	1.414297	35.33	Up
KRT17P1	.04535875	0.5046	-2.00131	-1.00984	14.26	Down
VWCE	.01862377	0.4364	2.352977	1.026929	12.17	Up
MDGA1	.01848599	0.5324	-2.35574	-1.25424	617.43	Down
SLC25A41	.0043863	0.3695	2.848955	1.052658	7.86	Up
KRT17P2	.00566897	0.4256	-2.76634	-1.17732	30.43	Down
ZFP57	.04006362	0.7857	2.053092	1.613086	62.96	Up
HCP5B	.02330042	0.4832	2.268473	1.096238	7.42	Up
GRIFIN	.02211928	0.451	-2.28831	-1.03193	10.77	Down
LOC100134317	.01013481	0.5727	-2.5712	-1.4725	17.36	Down
LINC02280	.00135034	0.3264	3.205061	1.046025	9.53	Up
LINC02362	.00152769	0.4144	-3.16937	-1.3133	12.89	Down
LOC107984703	.03552985	0.5058	2.102267	1.063333	115.55	Up

ADRA2A = adrenergic receptor alpha 2A, CA1 = carbonic anhydrase 1, CHD5 = chromodomain helicase DNA binding protein 5, CLDN9 = claudin 9, ITGAD = integrin alpha D, LC = long COVID, LfcSE = logfoldchange Standard Error, Log2FC = log2 fold change, m7G = N7-methylguanosine, Stat = statistic, TNFRSF17 = tumor necrosis factor receptor superfamily member 17.

### 3.3. PPI network construction and enrichment analyses

**Figure 4.**
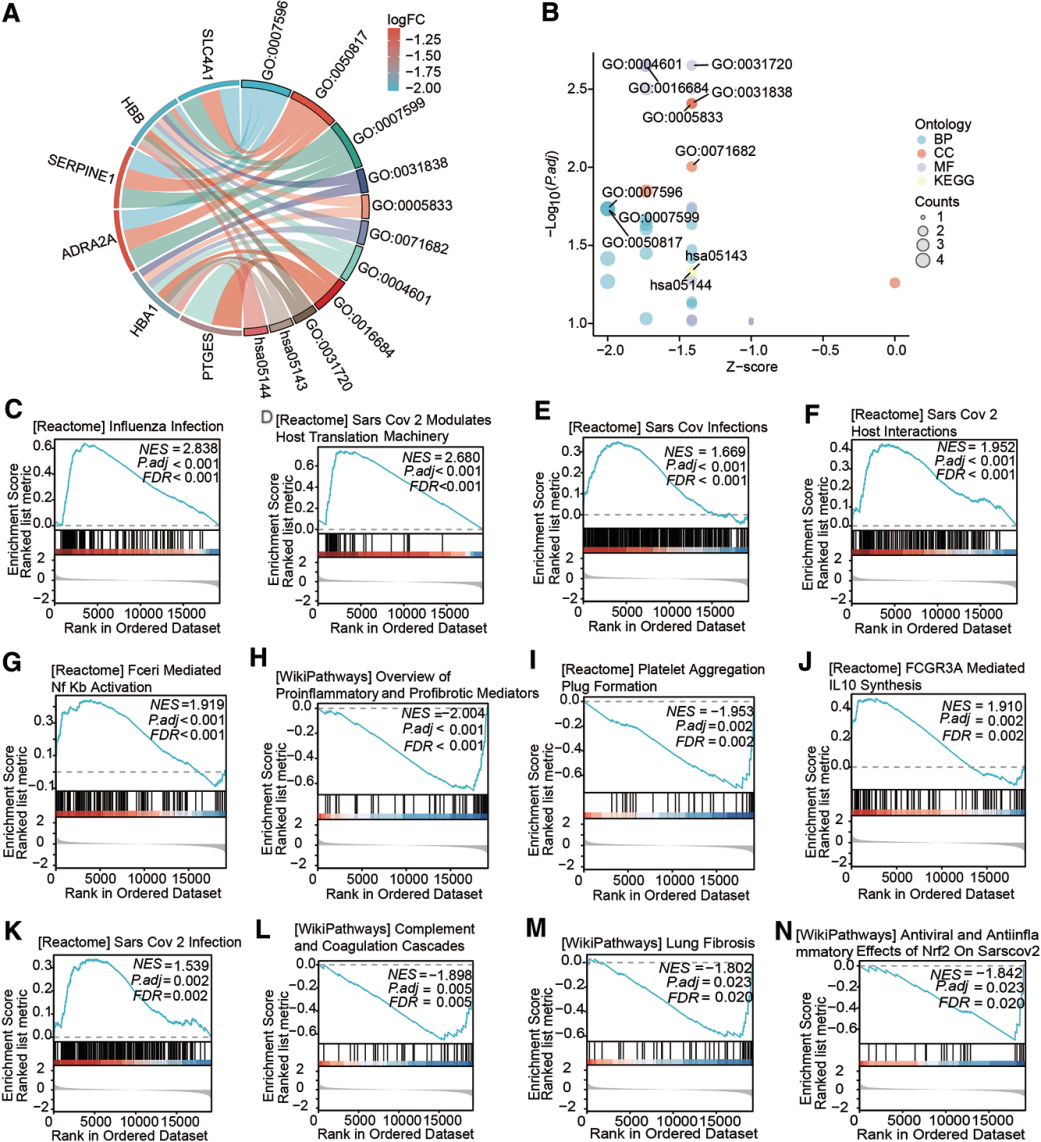
Enrichment analyses of crucial regulatory factors in m7G methylation among LC patients. (A) GO and (B) KEGG enrichment analyses; (C–N) GSEA enrichment analyses of crucial regulatory factors in m7G methylation among LC patients. GO = Gene Ontology, GSEA = gene set enrichment analysis, KEGG = Kyoto Encyclopedia of Genes and Genomes, LC = long COVID, m7G = N7-methylguanosine.

To elucidate the interactions among differentially expressed key regulatory factors of m7G methylation in patients with LC, we conducted a PPI analysis (Figure S1, Supplemental Digital Content, https://links.lww.com/MD/P803 and Table S1, Supplemental Digital Content, https://links.lww.com/MD/P804). To discern the potential biological functions associated with these differentially expressed key regulatory factors of m7G methylation in LC patients, we performed GO and KEGG enrichment analyses using R software. The outcomes unveiled the most significant GO-enriched terms, encompassing processes like blood coagulation and coagulation (biological processes), cellular components such as hemoglobin complex and endocytic vesicle lumen, and molecular functions including peroxidase activity and haptoglobin binding (Fig. 4A and B and Table S2, Supplemental Digital Content, https://links.lww.com/MD/P804). In the context of GSEA, the differentially expressed autophagy-related genes were primarily implicated in processes like Influenza infection, SARS-COV-2 modulates host translation machinery, FceRI-mediated NF-κB activation, WP overview of proinflammatory and profibrotic mediators, and platelet aggregation plug formation (Fig. 4C–N).

### 3.4. Correlation analysis of key genes and immunoinfiltrating organism phenotypes

**Figure 5.**
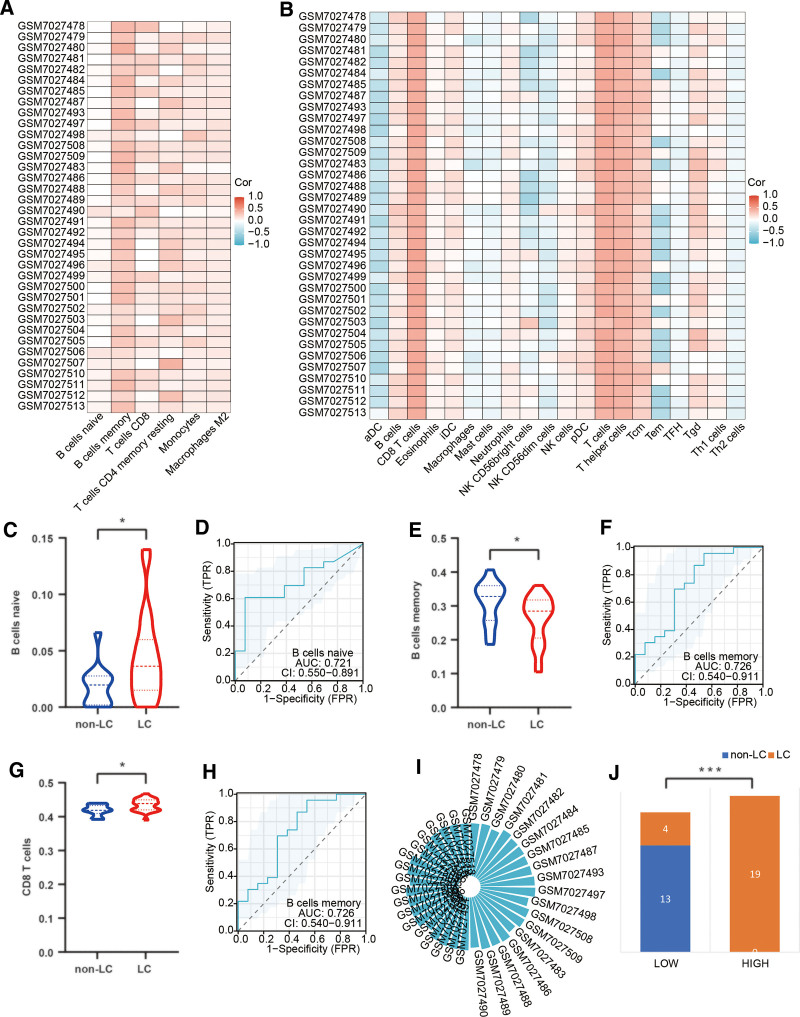
Correlation analysis of key genes and immunoinfiltrating organism phenotypes. (A) Correlation analysis using the CIBERSORT method. (B) Correlation analysis using the GSEA method. (C, D) Differential analysis and diagnostic efficacy of naive B cells between the 2 groups. (E, F) Differential analysis and diagnostic efficacy of memory B cells between the 2 groups. (G, H) Differential analysis and diagnostic efficacy of CD8 T cells between the 2 groups. (I) Immune cell scores analysis for crucial regulatory factors in m7G methylation among LC patients. (J) Differential analysis of immune cell scores between the 2 groups. GSEA = gene set enrichment analysis, LC = long COVID, m7G = N7-methylguanosine.

We conducted an analysis of immune cell populations (depicted in Fig. 5A and B) to examine the key regulatory factors associated with m7G methylation in patients suffering from LC, employing the CIBERSORT and GSEA methodologies. Within the context of key regulatory factors governing m7G methylation in LC patients, Naive B cells exhibited a significant increase in comparison to the control group, manifesting statistically substantial distinctions (as illustrated in Fig. 5C). Furthermore, this variation demonstrated noteworthy diagnostic efficacy, as illustrated in Figure 5D. Conversely, the subset of memory B cells which falls within the realm of key regulatory factors of m7G methylation in LC, demonstrated a marked reduction in comparison to the control group, and notably presented significant diagnostic efficiency, as highlighted in Figure 5E and F. We also observed statistically significant distinctions in CD8 T cells between the 2 groups, as depicted in Figure 5G and H. Employing the Estimation method, we assessed the immune cell scores within the key regulatory factors governing m7G methylation in LC patients, as presented in Figure 5I. These scores were categorized into high and low expression groups based on the median, revealing that the immune cell scores in the LC group were substantially higher than those in the control group, as portrayed in Figure 5J.

### 3.5. Validation and ROC curve of key genes

**Figure 6.**
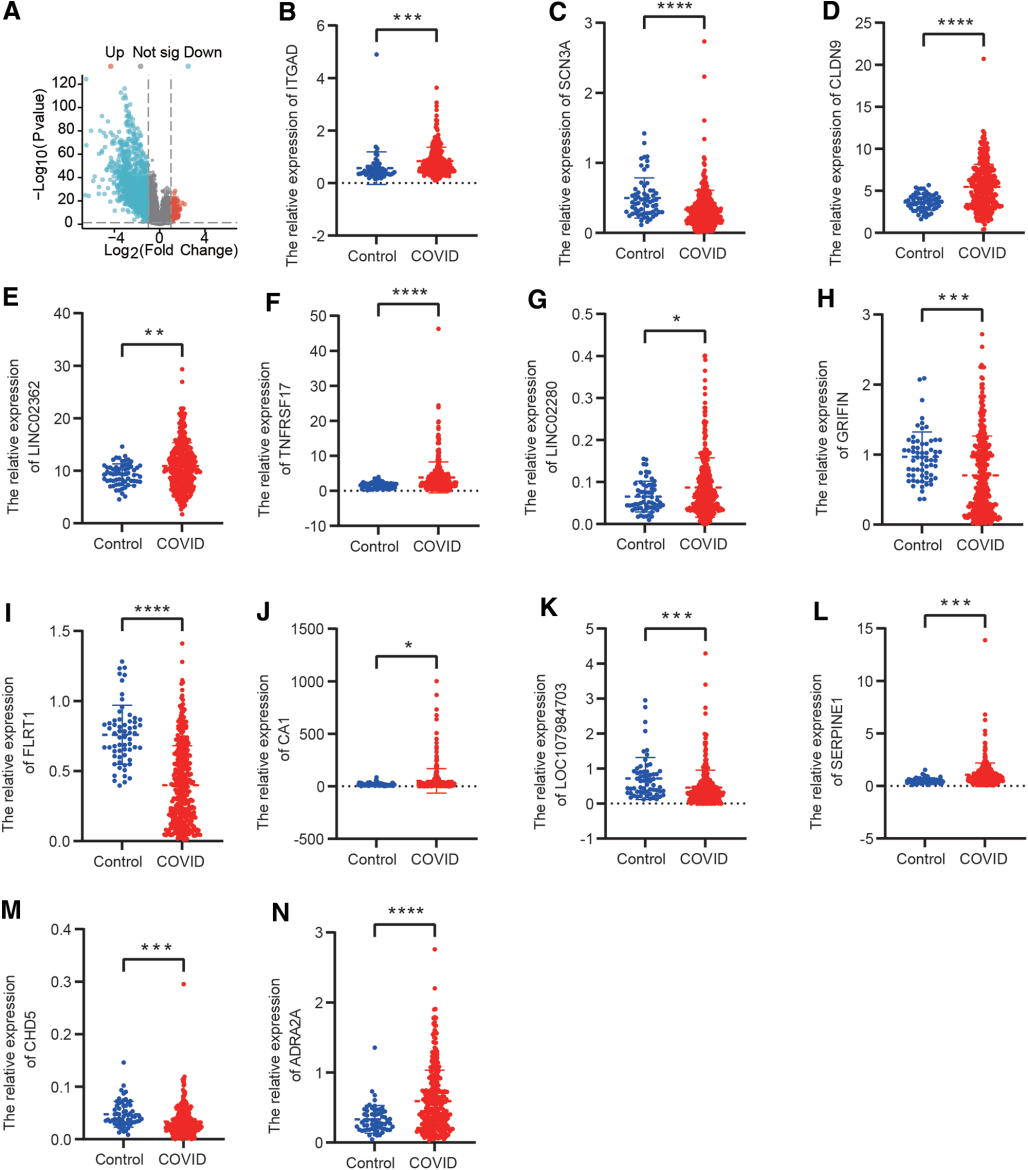
Validation of key regulatory factors in m7G methylation among LC patients. (A) Depiction of differential gene expression between the 2 groups illustrated in a volcano plot. (B–N) Differential expression analysis of the 13 key regulatory factors in m7G methylation among LC patients across the 2 groups. LC = long COVID, m7G = N7-methylguanosine.

To evaluate the robustness of the expression patterns of these pivotal genes, we conducted a validation using an external dataset, GSE217948. Initially, we conducted an analysis of the differentially expressed genes in this dataset, visually depicted in Figure 6A through a volcano plot. Subsequently, it was observed that 8 genes displayed a significant upregulation in expression within the COVID group, while 5 genes exhibited down-regulated expression, as shown in Figure 6B–N. Moreover, we performed ROC curve analysis using the validation dataset, revealing that these aforementioned 13 genes demonstrated admirable sensitivity and specificity. These distinctions are statistically significant, as illustrated in Figure S2A–M, Supplemental Digital Content, https://links.lww.com/MD/P803.

### 3.6. Development of the prediction model

**Figure 7.**
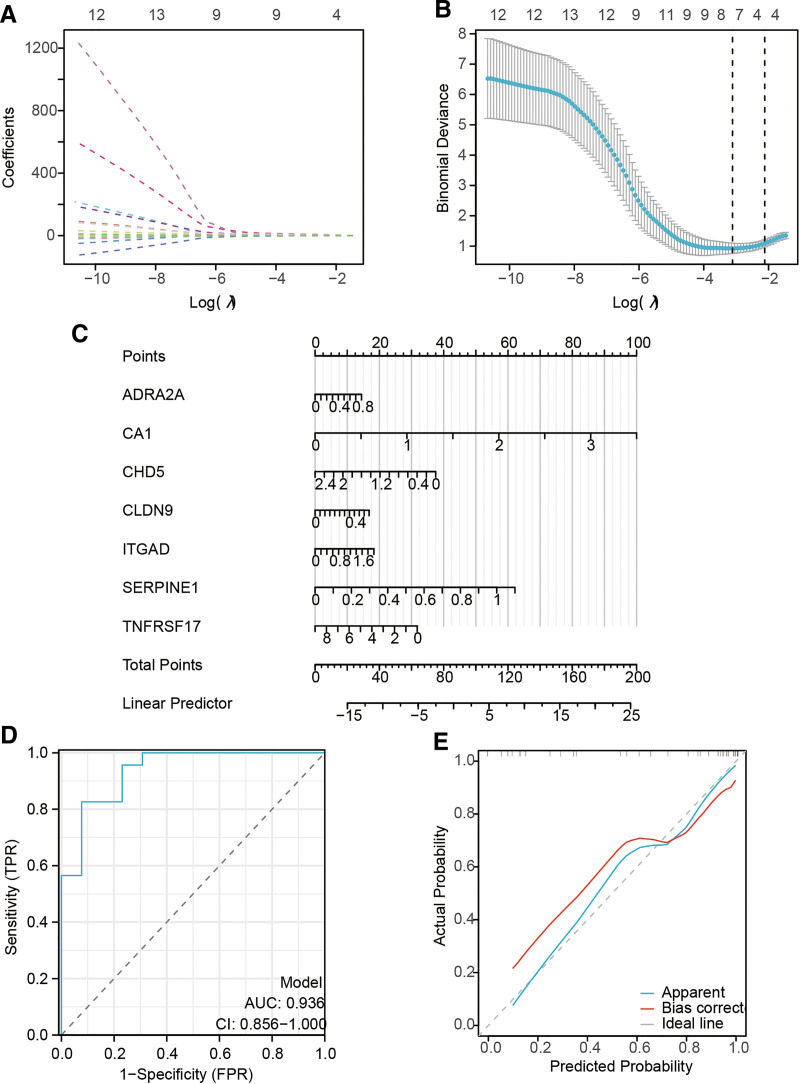
Development of the prediction model. (A, B) Utilization of the LASSO regression model for the identification of potential predictive factors. (C) Presentation of the prediction model via a nomogram. (D, E) Assessment of the model’s performance through ROC curves and calibration curves. LASSO = Least absolute shrinkage and selection operator, ROC = receiver operating characteristic.

Among the 13 pivotal genes, we discerned 7 genes utilizing the least absolute shrinkage and selection operator regression model. These chosen predictor variables all exhibit non-zero coefficients in the regression model, affirming their status as the optimal combination. The genes include adrenergic receptor alpha 2A, carbonic anhydrase 1, chromodomain helicase DNA binding protein 5, claudin 9, integrin alpha D, SERPINE1, tumor necrosis factor receptor superfamily member 17 (depicted in Fig. 7A and B and Table 2). Subsequently, we utilized these 7 genes to formulate a logistic regression model. To augment the model’s visual representation, we conceived a nomogram (Fig. 7C). The precision of this model was evaluated through ROC curves and calibration curves, unveiling a C-index of 0.936 (95% CI: 0.856–1.000) (Fig. 7D and E). Moreover, employing decision curve analysis on the nomogram for clinical applicability, the findings suggest substantial clinical efficacy of the model (Figure S3, Supplemental Digital Content, https://links.lww.com/MD/P803).

**Table 2:** The delineation of 7 genes identified via the LASSO regression model, which are subsequently employed in the development of the predictive model.

No.	Gene symbol	Official full name	Also known as
1	ADRA2A	Adrenoceptor alpha 2A	ADRA2; ADRAR; ZNF32; ADRA2R; ALPHA2AAR
2	CA1	Carbonic anhydrase 1	CAB; CA-I; Car1; HEL-S-11
3	CHD5	Chromodomain helicase DNA binding protein 5	CHD5; PMNDS
4	CLDN9	Claudin 9	DFNB116
5	ITGAD	Integrin subunit alpha D	ADB2; CD11D
6	SERPINE1	Serpin family E member 1	PAI; PAI1; PAI-1; PLANH1
7	TNFRSF17	TNF receptor superfamily member 17	BCM; BCMA; CD269; TNFRSF13A

ADRA2A = adrenergic receptor alpha 2A, CA1 = carbonic anhydrase 1, CHD5 = chromodomain helicase DNA binding protein 5, CLDN9 = claudin 9, ITGAD = integrin alpha D, LASSO = Least absolute shrinkage and selection operator, TNFRSF17 = tumor necrosis factor receptor superfamily member 17.

## 4. Discussion

This study represents the inaugural exposition of the expression patterns of m7G methylation regulatory factors in LC patients. Additionally, it unveils a potential correlation between the etiology of LC and the biological immune infiltration phenotype subsequent to m7G methylation modifications. This revelation provides an innovative vantage point for delving into the mechanistic underpinnings of LC. Moreover, we have devised the inaugural predictive model for LC, rooted in m7G methylation, thereby supplying a practical instrument for the early detection of LC patients in a clinical setting. While traditional method validation continues to be essential, future diagnostic approaches for complex diseases like LC may prioritize the characterization of predictive value, sensitivity, and specificity over the validation of analytical methods themselves. This shift reflects a growing emphasis on clinical relevance and real-world applicability in diagnostic models. Additionally, this study focused on evaluating the predictive value of m7G methylation in long COVID. However, given the diversity of other viral infections, future research should further assess the specificity and positive predictive value of this biomarker in these infections. This is crucial for confirming the broad applicability of the marker and its clinical potential.

Presently, an increasing body of evidence underscores the intricate relationship between methylation modifications and viral infection and replication. Take m6A, for instance. In the case of the influenza virus, posttranscriptional methylation occurs at internal adenosine residues, giving rise to m6A. To discern the distribution of m6A within various influenza virus mRNAs, Narayan P et al purified 6 of these mRNAs via hybrid selection, subjected them to nuclease digestion, and scrutinized their methylation patterns using high-pressure liquid chromatography.^[25]^ A study by Courtney DG et al has shed light on the impact of m6A incorporation within the context of influenza A virus (IAV).^[26]^ It was revealed that a global reduction in m6A incorporation coincides with a decrease in IAV gene expression and replication. Conversely, augmenting the “reader” protein YTHDF2 amplifies these pivotal viral processes. Another study unveiled an interaction between YTHDC1, an m6A reader protein, and the influenza A virus NS1 protein.^[27]^ This interaction was observed to exert influence on the splicing of viral mRNA, resulting in an upregulation of YTHDC1 levels following IAV infection. This interaction, in turn, led to the suppression of NS splicing at the NS 3’ splicing site, ultimately promoting IAV replication and pathogenicity both in vitro and in vivo. Kim D et al have documented the presence of methylation modifications in COVID-19 patients.^[28]^ Employing 2 complementary sequencing techniques, they offer a comprehensive depiction of the transcriptome and epitranscriptome of SARS-CoV-2. Furthermore, nanopore direct RNA sequencing reveals a minimum of 41 RNA modification sites on viral transcripts, with the most frequent motif identified as AAGAA. Altered RNAs exhibit shorter poly(A) tails compared to their unmodified counterparts, suggesting a potential connection between the modification and the 3’ tail. Currently, the foremost challenge we face pertains to the perplexing issue of LC.^[29,30]^ It is imperative to conduct further research to unravel the intricate interactions between m7G-modified viral RNA and host factors within LC patients. In our study, following stringent selection procedures, we have pinpointed 30 crucial regulatory factors associated with m7G methylation in LC patients. These genes hold significant promise as groundbreaking biomarkers for LC. By manipulating m7G modifications to modulate the expression of downstream pivotal target genes, these identified genes present a potential avenue for therapeutic strategies aimed at mitigating the effects of LC.

We conducted an in-depth enrichment analysis focusing on the pivotal regulatory factors associated with m7G methylation in the previously identified LC patients. Our findings revealed a notable intersection of enriched pathways with direct relevance to COVID, encompassing categories such as “SARS CoV 2 modulates host translation machinery,” “SARS CoV infections,” “SARS CoV 2 host interactions,” and “SARS CoV 2 infection.” This collective evidence underscores the significant role of m7G methylation modification in both the onset and progression of LC. Furthermore, our analysis highlighted the enrichment of 2 specific signaling pathways closely tied to pulmonary fibrosis: “lung fibrosis” and “overview of proinflammatory and profibrotic mediators.” It is important to note that numerous epidemiological studies have consistently pointed out that SARS-CoV-2 infection, particularly in the context of the COVID-19 pandemic, is associated with the potential development of severe pulmonary fibrotic consequences.^[31–33]^ Given these findings, it is reasonable to posit that targeted modulation of the identified m7G methylation regulatory factors could play a pivotal role in preventing or mitigating the onset of pulmonary fibrosis subsequent to COVID-19 infection. Moreover, our analysis unveiled the enrichment of 2 signaling pathways associated with coagulation disorders: “platelet aggregation plug formation” and “complement and coagulation cascades.” Multiple studies have unequivocally established that coagulation dysfunction constitutes a prominent characteristic of LC.^[34,35]^ The underlying mechanisms likely involve intricate interactions with the vascular endothelium, triggering persistent thromboinflammatory endotheliitis accompanied by systemic hypercoagulability. Consequently, this process fosters the formation of microclots resembling fibrin, excessive activation of platelets, and endothelial dysfunction, culminating in a spectrum of coagulopathic complications.^[36,37]^ Notably, there are currently no established treatments for these coagulation abnormalities, underscoring the urgent need for innovative therapeutic approaches. Our enrichment analysis outcomes strongly suggest that addressing coagulation dysfunction in the context of LC, through the lens of m7G methylation modifications, holds substantial promise and warrants further exploration.

The exaggerated and robust inflammatory response, commonly referred to as “hypercytokinemia” or “cytokine storm,” entails the excessive production and release of more than 150 inflammatory cytokines and chemical mediators by a diverse array of immune and non-immune cells.^[38]^ This response culminates in tissue damage, even though the specific triggers of this disproportionate immune reaction remain enigmatic. Myeloid cells, which encompass monocytes, macrophages, dendritic cells, and granulocytes (such as neutrophils, eosinophils, and basophils), assume a pivotal role in both mounting effective immune responses against viruses and in the initiation, propagation, and amplification of hypercytokinemia.^[38,39]^ Furthermore, research has elucidated a close interrelation between SARS-CoV-2 infection, cellular immune responses, and methylation modifications. For instance, many studies have shed light on the role of viral RNA modification, specifically m6A, as a critical signal for the innate immune system to differentiate self from non-self RNAs, thereby facilitating viral replication and gene expression.^[40–42]^ Additionally, their findings suggest that manipulating viral RNA to reduce m6A methylation could present a promising avenue in the development of live attenuated vaccines, as it has the potential to enhance the interferon response and adaptive immunity. Consequently, acquiring a comprehensive understanding of the immune characteristics exhibited by LC patients assumes paramount importance in unraveling the pathogenesis of SARS-CoV-2 and devising effective therapeutic strategies. Our enrichment results reveal that 4 immune-related pathways, namely “FceRI-mediated NF-κB activation,” “FCGR3A mediated IL10 synthesis,” “antiviral and anti-inflammatory effects of NRF2 ON SARS-CoV-2 pathway,” and “oxidative damage response,” are associated with m7G methylation modifications in LC patients. Subsequently, our immune infiltration analysis also reveals a correlation between key regulatory factors of m7G methylation and immune cell populations and immune cell scoring in LC patients. For instance, we observed a marked reduction in a subset of memory B cells which is among the essential regulatory factors of m7G methylation in LC, when compared to the control group. The study by Newell KL et al revealed that in convalescent COVID-19 patients, the presence of memory B cells in the peripheral blood exhibited an inverse correlation with the duration of symptoms.^[43]^ These memory B cells encompassed various subsets, including classical CD24 + class-switched, activated CD24-negative, and natural unswitched CD27 + IgD + IgM + subsets. Their frequency demonstrated a direct correlation with the levels of antibodies against the SARS-CoV-2 spike protein receptor binding domain, with a particular emphasis on IgM + memory B cells. Notably, IgM + memory B cells remained relatively stable and appeared to be crucial for establishing immunity against SARS-CoV-2. Therefore, it is plausible that targeting genes regulated by m7G methylation modifications could play a pivotal role in suppressing the persistence of LC from an immunological perspective.

Undoubtedly, this study is not devoid of limitations. We diligently screened the key regulatory factors of m7G methylation in LC patients and scrutinized the expression patterns of methylation modifications. Additionally, we subjected these pivotal factors to external validation. Nevertheless, it is imperative to acknowledge that our research represents an initial analysis. The specific conclusions and underlying mechanisms demand further validation through an extensive range of in vitro and in vivo experiments. Moreover, despite our establishment of a predictive model with commendable diagnostic efficacy, it is crucial to note that this model was developed using data from a single center. Consequently, its ability to be extrapolated is inherently restricted. Its effectiveness in the early diagnosis of LC still necessitates further validation and assessment within diverse, multicenter cohorts.

## 5. Conclusion

In summary, we have undertaken an initial investigation into the expression patterns of pivotal regulatory factors associated with m7G methylation in individuals with LC. Moreover, we have constructed a clinical predictive model grounded in genes subject to methylation modulation. It is crucial to emphasize that the enduring global repercussions of LC persist, and its underlying mechanisms may encompass a plethora of biological phenotypes, rendering it profoundly intricate. Hence, it necessitates further comprehensive scrutiny and exploration.

## Acknowledgments

This study was supported by Xinjiang Uygur Autonomous Region Natural Science Foundation (2023D01C140).

## Author contributions

**Conceptualization:** Wenmei Bai.

**Formal analysis:** Wenmei Bai, Fengsen Li.

**Funding acquisition:** Fengsen Li.

**Investigation:** Wenmei Bai, Fengsen Li.

**Methodology:** Wenmei Bai, Fengsen Li.

**Project administration:** Wenmei Bai, Fengsen Li.

**Resources:** Wenmei Bai, Fengsen Li.

**Software:** Wenmei Bai, Fengsen Li.

**Supervision:** Wenmei Bai.

**Validation:** Wenmei Bai.

**Visualization:** Wenmei Bai.

**Writing – original draft:** Wenmei Bai, Fengsen Li.

**Writing – review & editing:** Wenmei Bai, Fengsen Li.

## Supplementary Material


