# Supplementary material for: Regulation of m7G methylation in long COVID: Expression profiles and early predictive value of key genes
Source: Medicine (Baltimore). 2025 Aug 29;104(35):e44209. doi: 10.1097/MD.0000000000044209 (PMC12401457; doi:10.1097/MD.0000000000044209)

Supplementary Figure 1. Protein-Protein Interaction Network of Key Genes.


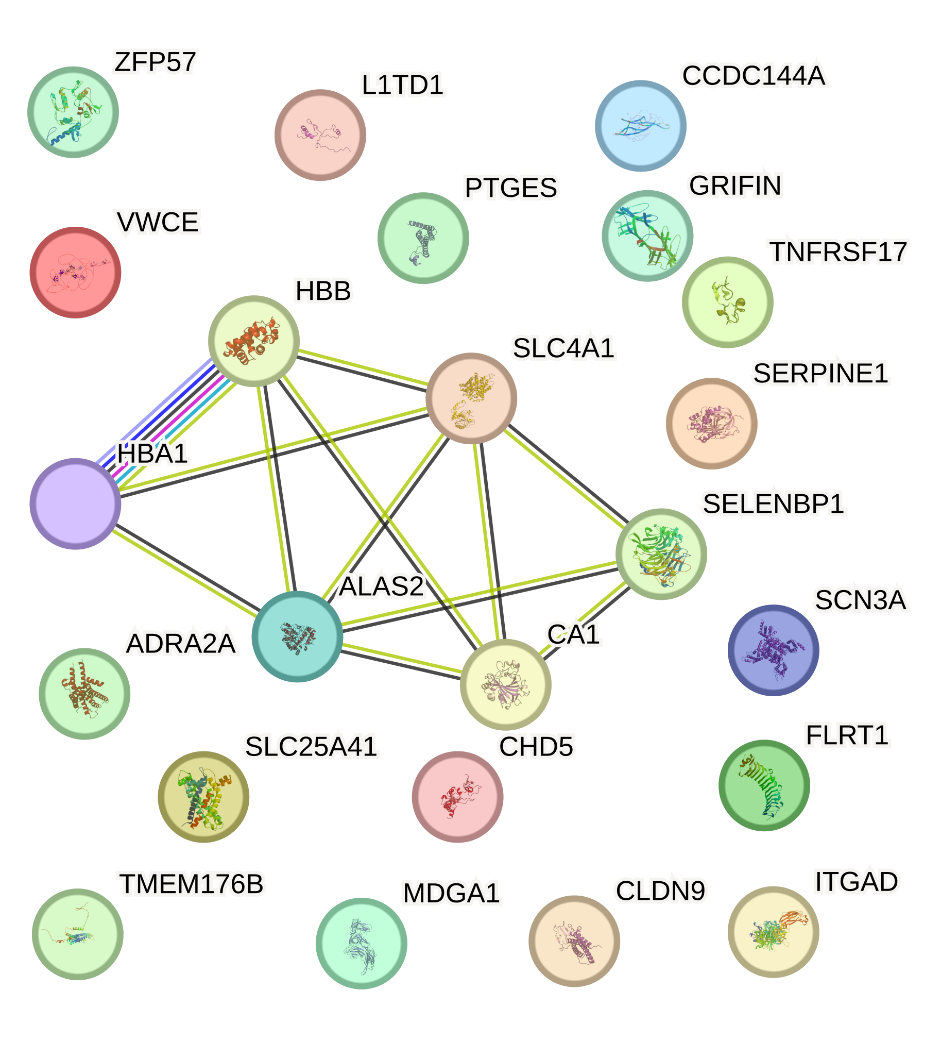


Supplementary Figure 2. ROC Curve Assessment of Diagnostic Performance for Key Genes.


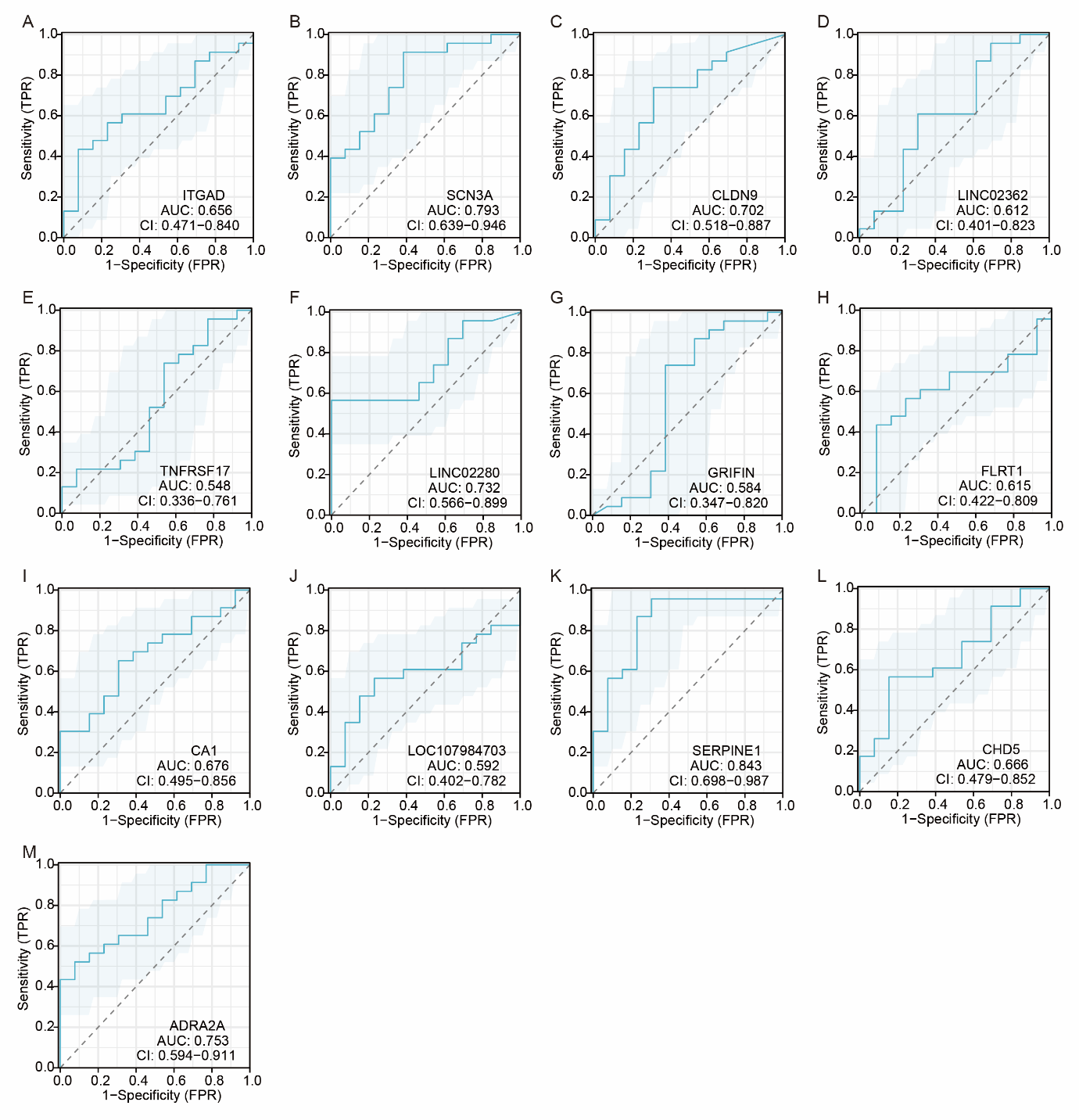


Supplementary Figure 3. Decision Curve Analysis for the Prediction Models Constructed from Key Genes.


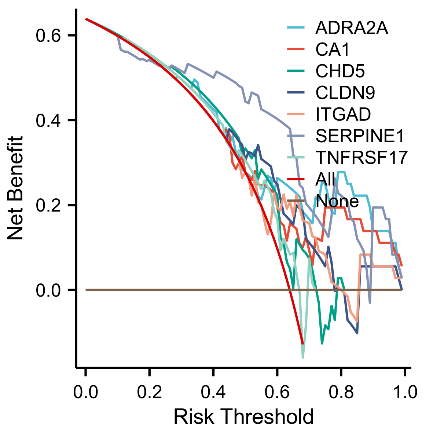

Supplement: Supplementary file 1 [file medi-104-e44209-s001.docx]
