# Supplementary material for: Regulation of m7G methylation in long COVID: Expression profiles and early predictive value of key genes
Source: Medicine (Baltimore). 2025 Aug 29;104(35):e44209. doi: 10.1097/MD.0000000000044209 (PMC12401457; doi:10.1097/MD.0000000000044209)
Supplement: Supplementary file 2 [file medi-104-e44209-s002.docx]

Supplementary Table 1. PPI network construction of Key Regulatory Factors in m7G Methylation Among LC Patients.

| node1 | node2 | node1_external_id | node2_external_id | coexpression | automated_textmining | combined_score |
| --- | --- | --- | --- | --- | --- | --- |
| HBA1 | HBB | ENSP00000322421 | ENSP00000494175 | 0.999 | 0.903 | 0.999 |
| SLC4A1 | ALAS2 | ENSP00000262418 | ENSP00000497236 | 0.680 | 0.631 | 0.876 |
| HBB | ALAS2 | ENSP00000494175 | ENSP00000497236 | 0.451 | 0.706 | 0.831 |
| SLC4A1 | CA1 | ENSP00000262418 | ENSP00000430656 | 0.785 | 0.212 | 0.824 |
| CA1 | ALAS2 | ENSP00000430656 | ENSP00000497236 | 0.769 | 0.210 | 0.81 |
| SELENBP1 | CA1 | ENSP00000397261 | ENSP00000430656 | 0.629 | 0.292 | 0.726 |
| HBA1 | ALAS2 | ENSP00000322421 | ENSP00000497236 | 0.500 | 0.457 | 0.717 |
| SLC4A1 | SELENBP1 | ENSP00000262418 | ENSP00000397261 | 0.514 | 0.280 | 0.635 |
| SELENBP1 | ALAS2 | ENSP00000397261 | ENSP00000497236 | 0.515 | 0.265 | 0.628 |
| SLC4A1 | HBB | ENSP00000262418 | ENSP00000494175 | 0.110 | 0.480 | 0.517 |
| SLC4A1 | HBA1 | ENSP00000262418 | ENSP00000322421 | 0.230 | 0.355 | 0.482 |
| CA1 | HBB | ENSP00000430656 | ENSP00000494175 | 0.140 | 0.350 | 0.418 |

Supplementary Table 2. GO and KEGG Enrichment Analyses of Crucial Regulatory Factors in m7G Methylation among LC Patients.

| Ontology | ID | Description | GeneRatio | BgRatio | P value | p.adjust |
| --- | --- | --- | --- | --- | --- | --- |
| BP | GO:0007596 | blood coagulation | 4/21 | 221/18800 | 9.51e-05 | 0.0184 |
| BP | GO:0050817 | coagulation | 4/21 | 226/18800 | 0.0001 | 0.0184 |
| BP | GO:0007599 | hemostasis | 4/21 | 227/18800 | 0.0001 | 0.0184 |
| BP | GO:0015671 | oxygen transport | 2/21 | 15/18800 | 0.0001 | 0.0184 |
| BP | GO:0098869 | cellular oxidant detoxification | 3/21 | 100/18800 | 0.0002 | 0.0216 |
| CC | GO:0031838 | haptoglobin-hemoglobin complex | 2/22 | 11/19594 | 6.58e-05 | 0.0039 |
| CC | GO:0005833 | hemoglobin complex | 2/22 | 12/19594 | 7.89e-05 | 0.0039 |
| CC | GO:0071682 | endocytic vesicle lumen | 2/22 | 23/19594 | 0.0003 | 0.0099 |
| CC | GO:0072562 | blood microparticle | 3/22 | 147/19594 | 0.0006 | 0.0142 |
| CC | GO:0098982 | GABA-ergic synapse | 2/22 | 70/19594 | 0.0028 | 0.0549 |
| MF | GO:0004601 | peroxidase activity | 3/23 | 52/18410 | 3.62e-05 | 0.0022 |
| MF | GO:0016684 | oxidoreductase activity, acting on peroxide as acceptor | 3/23 | 56/18410 | 4.52e-05 | 0.0022 |
| MF | GO:0031720 | haptoglobin binding | 2/23 | 10/18410 | 6.68e-05 | 0.0022 |
| MF | GO:0005344 | oxygen carrier activity | 2/23 | 14/18410 | 0.0001 | 0.0031 |
| MF | GO:0016209 | antioxidant activity | 3/23 | 85/18410 | 0.0002 | 0.0031 |
| KEGG | hsa05143 | African trypanosomiasis | 2/14 | 37/8164 | 0.0018 | 0.0463 |
| KEGG | hsa05144 | Malaria | 2/14 | 50/8164 | 0.0032 | 0.0463 |
